# Supplementary material for: Functional large-conductance calcium and voltage-gated potassium channels in extracellular vesicles act as gatekeepers of structural and functional integrity
Source: Nat Commun. 2025 Jan 2;16:42. doi: 10.1038/s41467-024-55379-4 (PMC11697022; doi:10.1038/s41467-024-55379-4)
Supplement: Supplementary file 1 — Supplementary Information [file 41467_2024_55379_MOESM1_ESM.pdf]

Functional large conductance calcium and voltage gated potassium channels in extracellular vesicles act as gatekeepers of structural and functional integrity.

Shridhar Sanghvi[1,2,*], Divya Sridharan[3,*], Parker Evans[4], Julie Dougherty[3], Kalina Szteyn[2], Denis Gabrilovich[1], Mayukha Dyta[2], Jessica Weist[3], Sandrine V. Pierre[5], Shubha Gururaja Rao[6], Dan R. Halm[7], Tingting Chen[8], Panagiotis S. Athanasopoulos[8], Amalia M. Dolga[8], Lianbo Yu[9], Mahmood Khan[3,#], Harpreet Singh[1,2,#]

[1]Department of Molecular Cellular and Developmental Biology, The Ohio State University, Columbus, OH, USA.

[2]Department of Physiology and Cell Biology, The Ohio State University Wexner Medical Center, Columbus, OH, USA.

[3]Department of Emergency Medicine, Division of Basic and Translation Research, The Ohio State University Wexner Medical Center, Columbus, OH, USA.

[4]Department of Mechanical and Aerospace Engineering, The Ohio State University, Columbus, OH, USA.

[5]Department of Biomedical Sciences, Joan C. Edwards School of Medicine, Marshall University, Huntington, WV, USA.

[6]Department of Pharmaceutical and Biomedical Sciences, The Raabe College of Pharmacy, Ohio Northern University, Ada, OH, USA.

[7]Department of Neuroscience, Cell Biology, and Physiology, Wright State University, Dayton, OH, USA.

[8]Department of Molecular Pharmacology, Groningen Research Institute of Pharmacy, University of Groningen, Groningen, NE.

[9]Department of Biomedical Informatics, The Ohio State University, Columbus, OH, USA.

*These authors have contributed equally to the manuscript.

#These are co-corresponding authors for the manuscript.

Corresponding authors:

Harpreet Singh, Ph.D. FAHA FCVS
Professor, Department of Physiology and Cell Biology
2166B, Graves Hall, 333 W 10th Avenue
The Ohio State University,
Columbus OH 43210 USA
Harpreet.singh@osumc.edu

Mahmood Khan, Ph.D., FAHA
Professor, Department of Emergency Medicine
Division of Basic and Translational Research
College of Medicine
The Ohio State University,
Columbus, OH 43210, USA
Mahmood.khan@osumc.edu

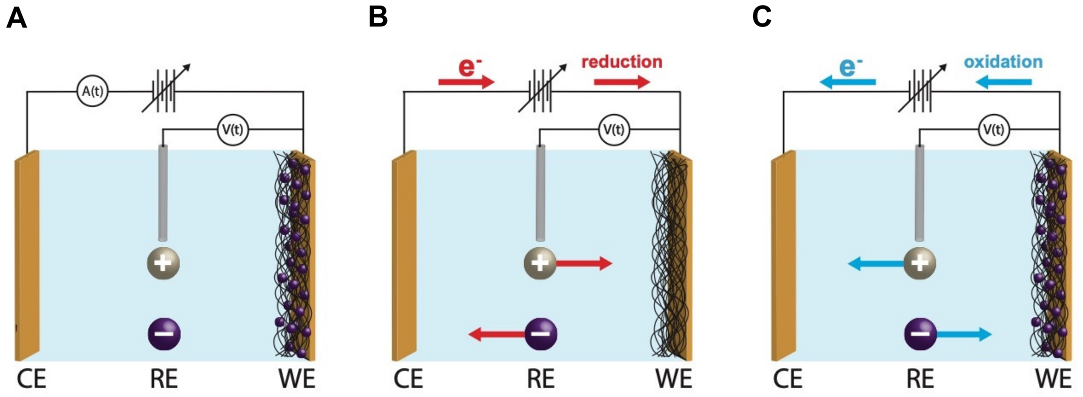

Supplementary Figure 1. Schematic diagram of ion transport in (**A**) Cl-doped polypyrrole in the absence of an applied potential, (**B**) Cl-doped polypyrrole under a reducing potential, and (**C**) Cl-doped polypyrrole under an oxidizing potential. The counter electrode is abbreviated (CE), the reference electrode (RE), and the working electrode (WE). Cations are depicted as grey spheres and anions are depicted as purple spheres.

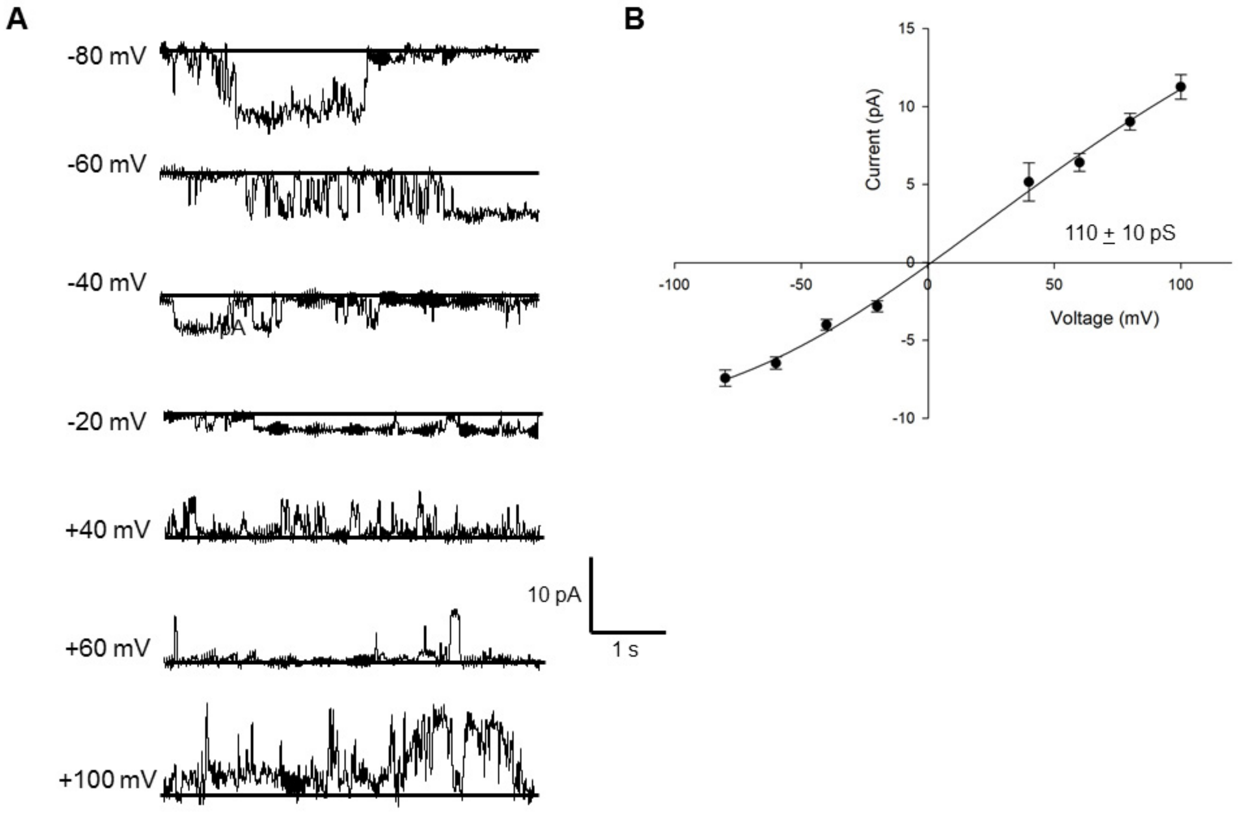

Supplementary Figure 2. A. Representative single channel traces for K^+ currents in EV membranes isolated from *Kcnma1*$^{-/-}$ mice. **B.** Current-voltage plot for K^+ channel showing 100±10 pS. The data represented as mean ± SD with 6 independent biological replicates.

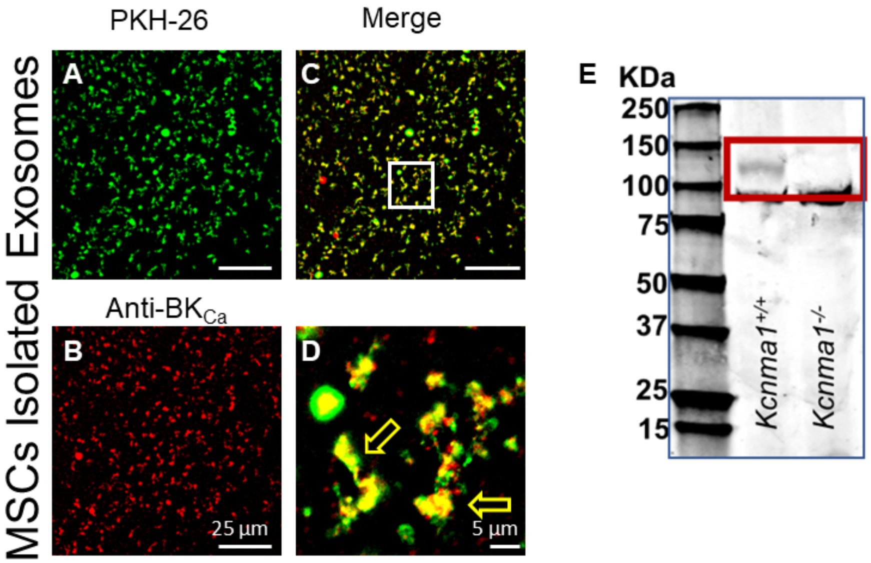

Supplementary Figure 3. Presence of BK$_{Ca}$ in EVs. A-D Human induced pluripotent stem cells derived Mesenchymal stem cells derived EVs were labelled with PKH67 (green), anti-BK$_{Ca}$ (red) and merged image shows localization. **E.** Anti-BK$_{Ca}$ antibody detect ~125 KDa band (red box) in *Kcnma1*$^{+/+}$ but not in *Kcnma1*$^{-/-}$ mice heart lysates.

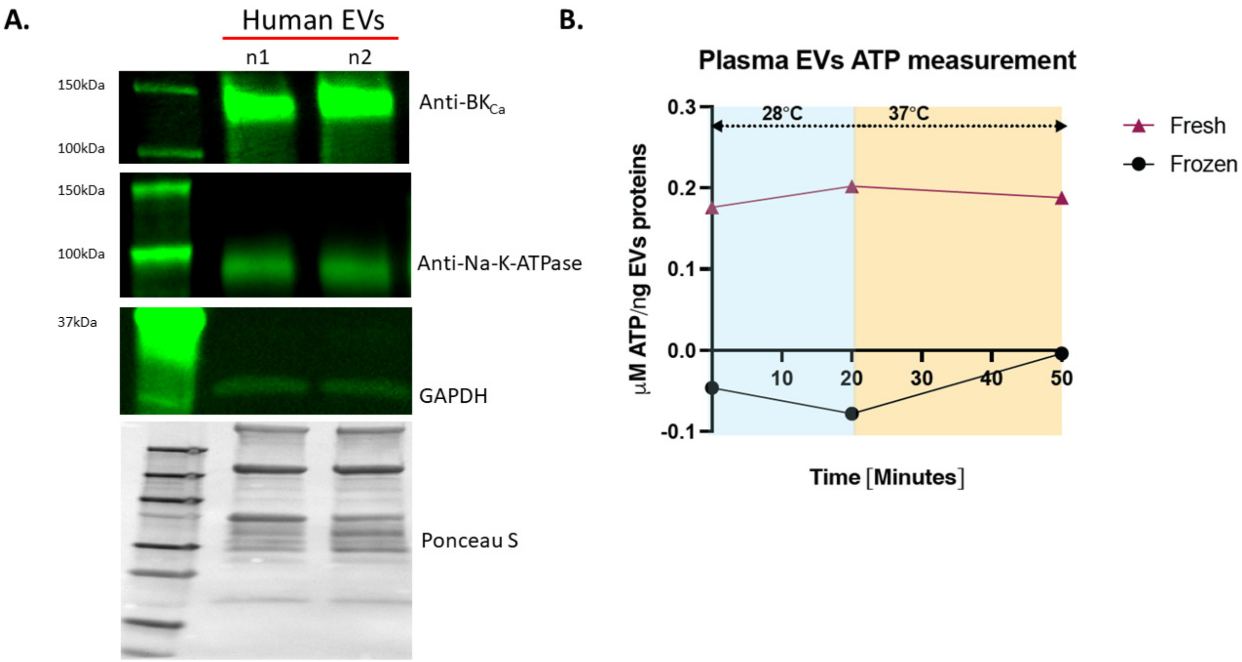

Supplementary Figure 4. ATP measurement in EVs. A. Human plasma derived EVs were positive for BK_{Ca} and Na-K-ATPase**. B.** The whole blood was collected from an healthy individual into an Ethylene Diamine Tetra Acetic acid (EDTA) tube, then it was processed to plasma within 15 min after withdrawal by two times centrifugation at 2,500g for 15 minutes, at room temperature. Izon 70nm qEV column (IZON) was used for isolating extracellular vesicles (EVs) from 500 µL fresh plasma. A total of 24 fractions, 500 µL of each, were collected from one isolation, in which fraction 7-11 are tested positive for EVs markers and used for further analysis. Plasma-derived EVs samples, both freshly isolated and stored at -80°C for more than one week, were used for measuring the ATP content. Protein concentrations of tested samples were measured by Micro BCA™ Protein Assay Kit (Thermo Fisher Scientific). EVs samples were 1:1 diluted in the assay buffer (450 mM sucrose, 88 mM KH_2PO_4, 25 mM magnesium acetate, and 12 mM EDTA, all reagents from Sigma-Aldrich). ATP standards and 80 ng/µL EVs samples were plated in White 96-Well Immuno Plates (Thermo Fisher Scientific), followed by adding detergent and substrate solution to each well. ATP standards, detergent, and substrate buffer were provided in Luminescent ATP Detection Assay Kit (abcam). Microplate was sealed and shaked for 5 minutes in an orbital shaker at 600- 700 rpm, then kept in dark for additional 10 minutes. Luminescence was read with a Synergy H1 Multi-Mode reader (Biotek, LA, USA) at 26°C, 37°C, and half an hour incubation at 37°C. ATP was detected in freshly isolated EVs but not in frozen samples.

Supplementary table 1 (Channels and transporters)

	Gene Name (Ion Channels)	Gene Symbol	Species
1	anoctamin 1, calcium activated chloride channel	Ano1	Mus musculus
2	acid-sensing (proton-gated) ion channel 3	Asic3	Mus musculus
3	calcium channel, voltage-dependent, N type, alpha 1B subunit	CACNA1B	Homo sapiens
4	calcium channel, voltage-dependent, R type, alpha 1E subunit	CACNA1E	Homo sapiens
5	calcium channel, voltage-dependent, L type, alpha 1S subunit	CACNA1S	Homo sapiens
6	calcium channel, voltage-dependent, alpha 2/delta subunit 1	CACNA2D1	Homo sapiens
7	calcium channel, voltage-dependent, alpha 2/delta subunit 2	CACNA2D2	Homo sapiens
8	calcium channel, voltage-dependent, alpha 2/delta subunit 4	CACNA2D4	Homo sapiens
9	calcium channel, voltage-dependent, beta 2 subunit	CACNB2	Bos taurus
10	calcium channel, voltage-dependent, gamma subunit 2	CACNG2	Bos taurus
11	chloride channel accessory 4	CLCA4	Homo sapiens
12	chloride channel, voltage-sensitive 2	Clcn2	Rattus norvegicus
13	chloride channel, voltage-sensitive 3	Clcn3	Rattus norvegicus
14	chloride channel, voltage-sensitive 3	CLCN3	Homo sapiens
15	chloride channel, voltage-sensitive 4	CLCN4	Homo sapiens
16	chloride channel, voltage-sensitive 5	CLCN5	Homo sapiens
17	chloride channel, voltage-sensitive 7	CLCN7	Homo sapiens
18	chloride intracellular channel 1	CLIC1	Homo sapiens
19	chloride intracellular channel 2	CLIC2	Homo sapiens
20	chloride intracellular channel 3	CLIC3	Homo sapiens
21	chloride intracellular channel 4	CLIC4	Homo sapiens
22	chloride intracellular channel 5	CLIC5	Homo sapiens
23	chloride intracellular channel 6	CLIC6	Homo sapiens
24	chloride channel, nucleotide-sensitive, 1A	CLNS1A	Homo sapiens
25	hyperpolarization activated cyclic nucleotide gated potassium channel 3	HCN3	Homo sapiens
26	potassium channel modulatory factor 1	KCMF1	Homo sapiens
27	potassium channel, voltage gated subfamily A regulatory beta subunit 2	KCNAB2	Homo sapiens
28	potassium channel, voltage gated Shaw-related subfamily C, member 2	KCNC2	Rattus norvegicus
29	potassium channel, voltage gated Shaw related subfamily C, member 3	KCNC3	Homo sapiens
30	potassium channel, voltage gated modifier subfamily G, member 1	KCNG1	Homo sapiens
31	potassium channel, voltage gated modifier subfamily G, member 2	KCNG2	Homo sapiens
32	potassium inwardly rectifying channel, subfamily J, member 11	KCNJ11	Mus musculus
33	potassium channel, inwardly rectifying subfamily J, member 12	KCNJ12	Homo sapiens

34	potassium inwardly-rectifying channel, subfamily J, member 15	KCNJ15	Mus musculus
35	potassium channel, inwardly rectifying subfamily J, member 2	KCNJ2	Homo sapiens
36	potassium inwardly-rectifying channel, subfamily J, member 3	KCNJ3	Mus musculus
37	potassium channel, calcium activated large conductance subfamily M alpha, member 1	KCNMA1	Homo sapiens
38	potassium large conductance calcium-activated channel, subfamily M, beta member 2	KCNMB2	Bos taurus
39	potassium channel, calcium activated intermediate/small conductance subfamily N alpha, member 4	KCNN4	Homo sapiens
40	potassium voltage-gated channel, modifier subfamily S, member 3	KCNS3	Homo sapiens
41	potassium channel, sodium activated subfamily T, member 1	KCNT1	Homo sapiens
42	potassium channel, subfamily V, member 2	KCNV2	Bos taurus
43	potassium channel tetramerization domain containing 1	KCTD1	Homo sapiens
44	potassium channel tetramerization domain containing 10	KCTD10	Homo sapiens
45	potassium channel tetramerization domain containing 12	KCTD12	Homo sapiens
46	potassium channel tetramerization domain containing 14	KCTD14	Homo sapiens
47	potassium channel tetramerization domain containing 15	KCTD15	Homo sapiens
48	potassium channel tetramerization domain containing 20	KCTD20	Homo sapiens
49	potassium channel tetramerization domain containing 21	KCTD21	Homo sapiens
50	purinergic receptor P2X, ligand gated ion channel, 1	P2RX1	Homo sapiens
51	purinergic receptor P2X, ligand gated ion channel, 4	P2RX4	Homo sapiens
52	purinergic receptor P2X, ligand gated ion channel, 5	P2RX5	Homo sapiens
53	purinergic receptor P2X, ligand-gated ion channel, 7	P2RX7	Mus musculus
54	piezo-type mechanosensitive ion channel component 1	Piezo1	Rattus norvegicus
55	similar to voltage-dependent anion channel 1	RGD1565338	Rattus norvegicus
56	sodium channel, voltage gated, type X alpha subunit	SCN10A	Homo sapiens
57	sodium channel, voltage gated, type XI alpha subunit	SCN11A	Homo sapiens
58	sodium channel, voltage-gated, type III, beta subunit	SCN3b	Rattus norvegicus
59	sodium channel, voltage gated, type V alpha subunit	SCN5A	Homo sapiens
60	sodium channel, non voltage gated 1 beta subunit	SCNN1B	Homo sapiens
61	transmembrane channel-like 6	TMC6	Homo sapiens
62	transmembrane channel-like 8	TMC8	Homo sapiens
63	two pore segment channel 1	TPCN1	Rattus norvegicus
64	transient receptor potential cation channel, subfamily C, member 4	TRPC4	Bos taurus
65	transient receptor potential cation channel, subfamily C, member 6	TRPC6	Homo sapiens
66	transient receptor potential cation channel, subfamily M, member 2	TRPM2	Mus musculus

67	transient receptor potential cation channel, subfamily M, member 4	TRPM4	Homo sapiens
68	transient receptor potential cation channel, subfamily V, member 2	TRPV2	Homo sapiens
69	transient receptor potential cation channel, subfamily V, member 4	TRPV4	Homo sapiens
70	voltage-dependent anion channel 1	VDAC1	Homo sapiens
71	voltage-dependent anion channel 2	VDAC2	Homo sapiens
72	voltage-dependent anion channel 3	VDAC3	Homo sapiens

	**Gene Name (Transporters)**	**Gene Symbol**	**Species**
1	solute carrier family 22 (organic cation transporter), member	2SLC22A2	Homo sapiens
2	arsA arsenite transporter, ATP-binding, homolog 1 (bacterial)	ASNA1	Homo sapiens
3	ATPase, Na+/K+ transporting, alpha 1 polypeptide	ATP1A1	Homo sapiens
4	ATPase, Na+/K+ transporting, alpha 2 polypeptide	ATP1A2	Homo sapiens
5	ATPase, Na+/K+ transporting, alpha 3 polypeptide	ATP1A3	Homo sapiens
6	ATPase, Na+/K+ transporting, beta 1 polypeptide	ATP1B1	Homo sapiens
7	ATPase, H+/K+ exchanging, alpha polypeptide	ATP4A	Homo sapiens
8	ATP synthase, H+ transporting, mitochondrial F1 complex, alpha subunit 1, cardiac muscle	ATP5A1	Homo sapiens
9	ATP synthase, H+ transporting, mitochondrial F1 complex, beta polypeptide	ATP5B	Homo sapiens
10	ATP synthase, H+ transporting, mitochondrial F1 complex, beta polypeptide	Atp5b	Rattus norvegicus
11	ATP synthase, H+ transporting, mitochondrial Fo complex, subunit E	ATP5I	Homo sapiens
12	ATP synthase, H+ transporting, mitochondrial Fo complex, subunit G	ATP5L	Homo sapiens
13	ATP synthase, H+ transporting, mitochondrial F1 complex, O subunit	ATP5O	Homo sapiens
14	ATPase, H+ transporting, lysosomal accessory protein 2	ATP6AP2	Homo sapiens
15	ATPase, H+ transporting, lysosomal 70kDa, V1 subunit A	ATP6V1A	Homo sapiens
16	ATPase, H+ transporting, lysosomal 42kDa, V1 subunit C2	ATP6V1C2	Homo sapiens
17	cystinosin, lysosomal cystine transporter	CTNS	Homo sapiens
18	ecto-NOX disulfide-thiol exchanger 1	Enox1	Rattus norvegicus
19	FXYD domain containing ion transport regulator 2	FXYD2	Homo sapiens
20	increased sodium tolerance 1 homolog (yeast)	IST1	Homo sapiens
21	magnesium transporter 1	MAGT1	Homo sapiens
22	mitochondrial calcium uniporter	MCU	Rattus norvegicus
23	membrane magnesium transporter 1	MMGT1	Homo sapiens

24	solute carrier family 10 (sodium/bile acid cotransporter), member 1	SLC10A1	Rattus norvegicus
25	solute carrier family 10 (sodium/bile acid cotransporter), member 1	SLC10A1	Rattus norvegicus
26	solute carrier family 12 (sodium/potassium/chloride transporter), member 1	SLC12A1	Homo sapiens
27	solute carrier family 12 (sodium/potassium/chloride transporter), member 2	SLC12A2	Homo sapiens
28	solute carrier family 12 (sodium/chloride transporter), member 3	SLC12A3	Homo sapiens
29	solute carrier family 12 (potassium/chloride transporter), member 4	SLC12A4	Homo sapiens
30	solute carrier family 12 (potassium/chloride transporter), member 5	SLC12A5	Bos taurus
31	solute carrier family 12 (potassium/chloride transporter), member 6	SLC12A6	Homo sapiens
32	solute carrier family 12 (potassium/chloride transporter), member 7	SLC12A7	Homo sapiens
33	solute carrier family 13 (sodium-dependent dicarboxylate transporter), member 2	SLC13A2	Bos taurus
34	solute carrier family 13 (sodium-dependent dicarboxylate transporter), member 3	SLC13A3	Homo sapiens
35	solute carrier family 15 (oligopeptide transporter), member 2	SLC15A2	Homo sapiens
36	solute carrier family 16 (monocarboxylate transporter), member 1	SLC16A1	Homo sapiens
37	solute carrier family 1 (neuronal/epithelial high affinity glutamate transporter, system Xag), member 1	SLC1A1	Homo sapiens
38	solute carrier family 1 (glial high affinity glutamate transporter), member 3	SLC1A3	Rattus norvegicus
39	solute carrier family 1 (neutral amino acid transporter), member 5	SLC1A5	Homo sapiens
40	kidney specific organic anion transporter	SLC21A4	Rattus norvegicus
41	solute carrier family 22 (organic anion/urate transporter), member 11	SLC22A11	Homo sapiens
42	solute carrier family 22 (organic anion/urate transporter), member 12	SLC22A12	Homo sapiens
43	solute carrier family 22 (organic anion/urate transporter), member 13	SLC22A13	Homo sapiens
44	solute carrier family 22 (organic cation transporter), member 2	SLC22A2	Homo sapiens
45	solute carrier family 22 (organic cation/carnitine transporter), member 5	SLC22A5	Homo sapiens
46	solute carrier family 22 (organic anion transporter), member 6	SLC22A6	Homo sapiens
47	solute carrier family 22 (organic anion transporter), member 8	SLC22A8	Homo sapiens
48	solute carrier family 24 (sodium/potassium/calcium exchanger), member 2	SLC24A2	Bos taurus

49	solute carrier family 25 (mitochondrial carrier; citrate transporter), member 1	SLC25A1	Homo sapiens
50	solute carrier family 25 (mitochondrial carrier; phosphate carrier), member 3	SLC25A3	Homo sapiens
51	solute carrier family 25 (mitochondrial iron transporter), member 37	SLC25A37	Rattus norvegicus
52	solute carrier family 26 (anion exchanger), member 11	SLC26A11	Homo sapiens
53	solute carrier family 26 (anion exchanger), member 2	SLC26A2	Homo sapiens
54	solute carrier family 26 (anion exchanger), member 4	SLC26A4	Homo sapiens
55	solute carrier family 26 (anion exchanger), member 6	SLC26A6	Homo sapiens
56	solute carrier family 26 (anion exchanger), member 8	SLC26A8	Bos taurus
57	solute carrier family 26 (anion exchanger), member 9	SLC26A9	Homo sapiens
58	solute carrier family 29 (equilibrative nucleoside transporter), member 1	SLC29A1	Homo sapiens
59	solute carrier family 2 (facilitated glucose transporter), member 1	SLC2A1	Homo sapiens
60	solute carrier family 2 (facilitated glucose transporter), member 12	SLC2A12	Homo sapiens
61	solute carrier family 2 (facilitated glucose transporter), member 14	SLC2A14	Homo sapiens
62	solute carrier family 2 (facilitated glucose transporter), member 2	SLC2A2	Rattus norvegicus
63	solute carrier family 2 (facilitated glucose transporter), member 3	SLC2A3	Homo sapiens
64	solute carrier family 2 (facilitated glucose transporter), member 4	SLC2A4	Rattus norvegicus
65	solute carrier family 2 (facilitated glucose/fructose transporter), member 5	SLC2A5	Homo sapiens
66	solute carrier family 34 (type II sodium/phosphate cotransporter), member 1	SLC34A1	Homo sapiens
67	solute carrier family 34 (type II sodium/phosphate cotransporter), member 2	SLC34A2	Homo sapiens
68	solute carrier family 35 (UDP-GlcNAc/UDP-glucose transporter), member D2	SLC35D2	Homo sapiens
69	solute carrier family 36 (proton/amino acid symporter), member 1	Slc36a1	Rattus norvegicus
70	solute carrier family 36 (proton/amino acid symporter), member 2	SLC36A2	Homo sapiens
71	solute carrier family 37 (glucose-6-phosphate transporter), member 2	SLC37A2	Homo sapiens
72	solute carrier family 3 (amino acid transporter heavy chain), member 1	SLC3A1	Homo sapiens
73	solute carrier family 3 (amino acid transporter heavy chain), member 2	SLC3A2	Homo sapiens
74	solute carrier family 40 (iron-regulated transporter), member 1	SLC40A1	Homo sapiens

75	solute carrier family 44 (choline transporter), member 2	SLC44A2	Homo sapiens
76	solute carrier family 4 (anion exchanger), member 1 (Diego blood group)	SLC4A1	Homo sapiens
77	solute carrier family 4, sodium borate transporter, member 11	SLC4A11	Homo sapiens
78	solute carrier family 4 (anion exchanger), member 2	SLC4A2	Homo sapiens
79	solute carrier family 4 (sodium bicarbonate cotransporter), member 4	SLC4A4	Homo sapiens
80	solute carrier family 4, sodium bicarbonate cotransporter, member 7	SLC4A7	Homo sapiens
81	solute carrier family 4, sodium bicarbonate cotransporter, member 8	SLC4A8	Homo sapiens
82	solute carrier family 5 (sodium/glucose cotransporter), member 1	SLC5A1	Homo sapiens
83	solute carrier family 5 (sodium/sugar cotransporter), member 10	SLC5A10	Homo sapiens
84	solute carrier family 5 (sodium/monocarboxylate cotransporter), member 12	SLC5A12	Homo sapiens
85	solute carrier family 5 (sodium/glucose cotransporter), member 2	SLC5A2	Homo sapiens
86	solute carrier family 5 (sodium/myo-inositol cotransporter), member 3	SLC5A3	Homo sapiens
87	solute carrier family 5 (sodium/iodide cotransporter), member 5	SLC5A5	Homo sapiens
88	solute carrier family 5 (sodium/multivitamin and iodide cotransporter), member 6	SLC5A6	Homo sapiens
89	solute carrier family 5 (sodium/monocarboxylate cotransporter), member 8	SLC5A8	Homo sapiens
90	solute carrier family 5 (sodium/sugar cotransporter), member 9	SLC5A9	Bos taurus
91	solute carrier family 6 (neurotransmitter transporter), member 13	SLC6A13	Homo sapiens
92	solute carrier family 6 (amino acid transporter), member 14	SLC6A14	Homo sapiens
93	solute carrier family 7 (anionic amino acid transporter light chain, xc- system), member 11	SLC7A11	Homo sapiens
94	solute carrier family 7 (amino acid transporter light chain, L system), member 5	SLC7A5	Homo sapiens
95	solute carrier family 8 (sodium/calcium exchanger), member 1	SLC8A1	Homo sapiens
96	solute carrier family 9, subfamily A (NHE1, cation proton antiporter 1), member 1	SLC9A1	Homo sapiens
97	solute carrier family 9, subfamily A (NHE3, cation proton antiporter 3), member 3	SLC9A3	Homo sapiens
98	solute carrier family 9, subfamily A (NHE3, cation proton antiporter 3), member 3 regulator 1	SLC9A3R1	Homo sapiens
99	solute carrier family 9, subfamily A (NHE3, cation proton antiporter 3), member 3 regulator 2	SLC9A3R2	Homo sapiens

100	solute carrier family 9, subfamily A (NHE4, cation proton antiporter 4), member 4	SLC9A4	Bos taurus
101	solute carrier family 9 (sodium/hydrogen exchanger), member 8	SLC9A8	Mus musculus
102	solute carrier family 9, subfamily A (NHE9, cation proton antiporter 9), member 9	SLC9A9	Homo sapiens
103	solute carrier organic anion transporter family, member 1a1	SLCO1A1	Rattus norvegicus
104	solute carrier organic anion transporter family, member 1A2	SLCO1A2	Bos taurus
105	solute carrier organic anion transporter family, member 3A1	SLCO3A1	Homo sapiens
106	solute carrier organic anion transporter family, member 4A1	SLCO4A1	Homo sapiens
107	solute carrier organic anion transporter family, member 4C1	SLCO4C1	Homo sapiens

REFERENCES AND NOTES

1 Dougherty, J. A. *et al.* Human Cardiac Progenitor Cells Enhance Exosome Release and Promote Angiogenesis Under Physoxia. *Front Cell Dev Biol* **8**, 130, doi:10.3389/fcell.2020.00130 (2020).

2 Gururaja Rao, S. *et al.* Identification and Characterization of a Bacterial Homolog of Chloride Intracellular Channel (CLIC) Protein. *Scientific reports* **7**, 8500, doi:10.1038/s41598-017-08742-z (2017).

3 Venugopal, V. & Sundaresan, V. B. Polypyrrole-based amperometric cation sensor with tunable sensitivity. *Journal of Intelligent Material Systems and Structures* **27**, 1702-1709, doi:10.1177/1045389X15604233 (2016).

4 Venugopal, V., Venkatesh, V., Northcutt, R. G., Maddox, J. & Sundaresan, V. B. Nanoscale polypyrrole sensors for near-field electrochemicalmeasurements. *Sensors and Actuators B: Chemical* **242**, 1193-1200, doi:10.1016/j.snb.2016.09.121 (2017).

5 Gupta, S., Otero, J. J., Sundaresan, V. B. & Czeisler, C. M. Near field non-invasive electrophysiology of retrotrapezoid nucleus using amperometric cation sensor. *Biosensors and Bioelectronics* **151**, 111975, doi:https://doi.org/10.1016/j.bios.2019.111975 (2020).

6 Venugopal, V., T., H., Venkatesh, V. & Sundaresan, V. B. Mass and charge density effects on the saturation kinetics of polypyrrole doped with dodecylbenzene sulfonate. *Journal of Intelligent Material Systems and Structures* **28**, 760-771, doi:10.1177/1045389X16657421 (2017).

7 Gallego-Perez, D. *et al.* Topical tissue nano-transfection mediates non-viral stroma reprogramming and rescue. *Nature nanotechnology* **12**, 974-979, doi:10.1038/nnano.2017.134 (2017).

8 Gao, M. *et al.* Direct observation of liquid crystals using cryo-TEM: specimen preparation and low-dose imaging. *Microsc Res Tech* **77**, 754-772, doi:10.1002/jemt.22397 (2014).

9 Singh, H. *et al.* mitoBKCa is encoded by the Kcnma1 gene, and a splicing sequence defines its mitochondrial location. *Proceedings of the National Academy of Sciences of the United States of America* **110**, 10836-10841, doi:10.1073/pnas.1302028110 (2013).

10 Sanghvi, S. *et al.* Inhibition of BK(Ca) channels protects neonatal hearts against myocardial ischemia and reperfusion injury. *Cell Death Discov* **8**, 175, doi:10.1038/s41420-022-00980-z (2022).

11 Zinchuk, V., Wu, Y., Grossenbacher-Zinchuk, O. & Stefani, E. Quantifying spatial correlations of fluorescent markers using enhanced background reduction with protein proximity index and correlation coefficient estimations. *Nature protocols* **6**, 1554-1567, doi:10.1038/nprot.2011.384 (2011).

12 Ponnalagu, D. *et al.* CLIC4 localizes to mitochondrial-associated membranes and mediates cardioprotection. *Sci Adv* **8**, eabo1244, doi:10.1126/sciadv.abo1244 (2022).
