# Supplementary material for: Functional large-conductance calcium and voltage-gated potassium channels in extracellular vesicles act as gatekeepers of structural and functional integrity
Source: Nat Commun. 2025 Jan 2;16:42. doi: 10.1038/s41467-024-55379-4 (PMC11697022; doi:10.1038/s41467-024-55379-4)
Supplement: Supplementary file 2 — Reporting Summary [file 41467_2024_55379_MOESM2_ESM.pdf]

nature portfolio

Corresponding author(s): Mahmood Khan and Harpreet Singh

Last updated by author(s): Dec 6, 2024

Reporting Summary

Nature Portfolio wishes to improve the reproducibility of the work that we publish. This form provides structure for consistency and transparency in reporting. For further information on Nature Portfolio policies, see our Editorial Policies and the Editorial Policy Checklist.

Statistics

For all statistical analyses, confirm that the following items are present in the figure legend, table legend, main text, or Methods section.

n/a	Confirmed	
☐	☒	The exact sample size (*n*) for each experimental group/condition, given as a discrete number and unit of measurement
☐	☒	A statement on whether measurements were taken from distinct samples or whether the same sample was measured repeatedly
☐	☒	The statistical test(s) used AND whether they are one- or two-sided<br>*Only common tests should be described solely by name; describe more complex techniques in the Methods section.*
☒	☐	A description of all covariates tested
☐	☒	A description of any assumptions or corrections, such as tests of normality and adjustment for multiple comparisons
☐	☒	A full description of the statistical parameters including central tendency (e.g. means) or other basic estimates (e.g. regression coefficient) AND variation (e.g. standard deviation) or associated estimates of uncertainty (e.g. confidence intervals)
☐	☒	For null hypothesis testing, the test statistic (e.g. *F*, *t*, *r*) with confidence intervals, effect sizes, degrees of freedom and *P* value noted<br>*Give P values as exact values whenever suitable.*
☒	☐	For Bayesian analysis, information on the choice of priors and Markov chain Monte Carlo settings
☒	☐	For hierarchical and complex designs, identification of the appropriate level for tests and full reporting of outcomes
☒	☐	Estimates of effect sizes (e.g. Cohen's *d*, Pearson's *r*), indicating how they were calculated

Our web collection on statistics for biologists contains articles on many of the points above.

Software and code

Policy information about availability of computer code

Data collection	The electrophysiology single channel EV recordings were conducted on bilayer clamp amplifier (BC-535, Warner Instruments) and data curation using Clampfit 10.7. The size analysis of EVs was performed on NanoSight NS300 (Malvern Panalytical, Malvern, United Kingdom) and the data was consolidated using the NTA version 3.3 (Malvern Panalytical, Malvern, United Kingdom). The multi electrode array (MEA) experiments were performed on AxIS Navigator version 2.0.4 platoform and the evaluation of the cardiac parameters were assessed using the Cardiac Analysis Tool version 3.1.8. All imaging experiments were performed on Nikon A1R high-resolution confocal microscopy. Echocardiography after ischemia-referpusion injury was evaluated on Vevo 3100 Imaging System (FUJIFILM VisualSonics Inc., Toronto, Canada) and cardiac function parameters were measured on Vevo labs version 5.7.1. miRNA content of EVs was assessed in triplicate samples according to manufacturers' protocol (NanoString Technologies, Inc. Seattle, USA) by using nCounter Human v3 miRNA Expression Assay Kit (Cat# GXA-MIR3-12).
Data analysis	Statistical and graphical analysis were performed using GraphPad Prism Version 10.3.1 and Microsoft excel. Confocal images and representation of blots were processed using ImageJ (National Institutes of Health, Bethesda, MD, USA).

For manuscripts utilizing custom algorithms or software that are central to the research but not yet described in published literature, software must be made available to editors and reviewers. We strongly encourage code deposition in a community repository (e.g. GitHub). See the Nature Portfolio guidelines for submitting code & software for further information.

Data

Policy information about availability of data

All manuscripts must include a data availability statement. This statement should provide the following information, where applicable:

- Accession codes, unique identifiers, or web links for publicly available datasets
- A description of any restrictions on data availability
- For clinical datasets or third party data, please ensure that the statement adheres to our policy

Source data and supplementary files are included with the manuscript.

Research involving human participants, their data, or biological material

Policy information about studies with human participants or human data. See also policy information about sex, gender (identity/presentation), and sexual orientation and race, ethnicity and racism.

Reporting on sex and gender	NA
Reporting on race, ethnicity, or other socially relevant groupings	NA
Population characteristics	NA
Recruitment	NA
Ethics oversight	NA

Note that full information on the approval of the study protocol must also be provided in the manuscript.

Field-specific reporting

Please select the one below that is the best fit for your research. If you are not sure, read the appropriate sections before making your selection.

☒ Life sciences ☐ Behavioural & social sciences ☐ Ecological, evolutionary & environmental sciences

For a reference copy of the document with all sections, see nature.com/documents/nr-reporting-summary-flat.pdf

Life sciences study design

All studies must disclose on these points even when the disclosure is negative.

Sample size	The in vivo sample sizes for this study were determined based on power calculations outlined in the IACUC animal protocol, ensuring they were adequate to achieve statistical significance. For in vitro experiments, all biological replicates were included, and sample sizes were selected based on our lab's rationale. Specifically, the aim was to achieve approximately 80% power to detect a 1.5-fold difference in standard deviations for the parameters of interest between two treatment groups.
Data exclusions	No data points were excluded in this study.
Replication	All the experiments were successfully reproduced atleast three times
Randomization	In this study experiments involving in vitro hiPSC-CMs, wells were randomly assigned to experimental groups to eliminate positional bias within the MEA plate. Experimental rigor was upheld by maintaining consistent culture conditions, adhering to standardized cell seeding protocols, and including replicates to enhance reproducibility and reduce technical variability. For in vivo experiments, mice were age- and weight-matched before being randomly allocated to experimental groups, which included sham and IR surgeries.
Blinding	The ischemia-reperfusion surgeries were conducted by a n investigator blinded to the identity of the intramyocardial injections to ensure procedural impartiality. Echocardiographic assessments and subsequent analyses were performed using randomly allocated, ear-tagged animals, with the investigator blinded to the treatment groups to eliminate bias. Likewise, all in vitro experiments and bioinformatic analyses were carried out under double blinded conditions to maintain objectivity and scientific rigor.

Reporting for specific materials, systems and methods

We require information from authors about some types of materials, experimental systems and methods used in many studies. Here, indicate whether each material, system or method listed is relevant to your study. If you are not sure if a list item applies to your research, read the appropriate section before selecting a response.

Materials & experimental systems

n/a	Involved in the study
☐	☒ Antibodies
☐	☒ Eukaryotic cell lines
☒	☐ Palaeontology and archaeology
☐	☒ Animals and other organisms
☒	☐ Clinical data
☒	☐ Dual use research of concern
☒	☐ Plants

Methods

n/a	Involved in the study
☒	☐ ChIP-seq
☒	☐ Flow cytometry
☒	☐ MRI-based neuroimaging

Antibodies

Antibodies used

Anti-BKCa (Alomone labs, #APC21)
Anti-Na-K-ATPase (Abcam, #ab7671)
Anti-TOMM20 (Thermo Fisher Scientific, #MA5-32148)
Anti-ALIX (Cell Signaling Technology, #2171S)
Anti-GAPDH (Cell Signaling Technology, #2118)
Anti-CD-81 (System Biosciences, #EXOAB-CD81A-1)
Anti-E-Cadherin (Thermo Fisher Scientific, #14-3249-82)

Validation

anti-BKCa (Alomone labs, #APC21), we have validated the antibody in the supplementary figure: https://www.alomone.com/p/anti-kca1-1-1097-1196/APC-021
anti-Na-K-ATPase (Abcam, #ab7671): https://www.abcam.com/en-us/products/primary-antibodies/alpha-1-sodium-potassium-atpase-antibody-4646-ab7671
anti-TOMM20 (Thermo Fisher Scientific, #MA5-32148): https://www.thermofisher.com/antibody/product/TOMM20-Antibody-clone-ST04-72-Recombinant-Monoclonal/MA5-32148
anti-ALIX (Cell Signaling Technology, #2171S): https://www.cellsignal.com/products/primary-antibodies/alix-3a9-mouse-mab/2171
anti-GAPDH (Cell Signaling Technology, #2118): https://www.cellsignal.com/products/primary-antibodies/gapdh-14c10-rabbit-mab/2118
anti-CD-81 (System Biosciences, #EXOAB-CD81A-1): https://www.systembio.com/products/exosome-research/exosome-detection/antibodies/exosome-marker/anti-cd81-antibody-with-goat-anti-rabbit-hrp-secondary-antibody
anti-E-Cadherin (Thermo Fisher Scientific, #14-3249-82): https://www.thermofisher.com/antibody/product/CD324-E-Cadherin-Antibody-clone-DECMA-1-Monoclonal/14-3249-82

Eukaryotic cell lines

Policy information about cell lines and Sex and Gender in Research

Cell line source(s)

Human induced pluripotent stem cells derived cardiomyocytes (hiPSC-CM; iCell cardiomyocytes) from FUJIFILM Cellular Dynamics, Inc were used in this study.

Authentication

Cell were purchased from FUJIFILM Cellular Dynamics, Inc.

Mycoplasma contamination

Cells were tested and free from mycoplasma contamination.

Commonly misidentified lines
(See ICLAC register)

No misidentified lines were used in this study.

Animals and other research organisms

Policy information about studies involving animals; ARRIVE guidelines recommended for reporting animal research, and Sex and Gender in Research

Laboratory animals

C57BL/6NCrL male mice three months of age were used in this study. The mice were housed in ventilated cages under controlled environmental conditions. The housing facility maintained a 12-hour light/dark cycle (lights on at 6 AM), with an ambient temperature of 20–24°C and relative humidity of 40–60%. Animals had ad libitum access to standard chow food and water unless otherwise specified.

Wild animals

This study does not involve any wild animals.

Reporting on sex

This study includes work on only with male C57BL/6NCrL mice.

Field-collected samples

This study doe not include any samples collected from the field.

Ethics oversight

All animal studies followed the Guide for the Care and Use of Laboratory Animals (NIH Publication, 8th Edition, 2011) and procedures

were approved by the Institutional Animal Care and Use Committee of The Ohio State University under protocol 2018A00000095 and human plasma were processed after the Human Research Ethics Board Approval from University of Groningen (Lifelines project number: OV21_00315).

Note that full information on the approval of the study protocol must also be provided in the manuscript.

Plants

Seed stocks

NA

Novel plant genotypes

NA

Authentication

NA
